# Interaction between Positive End Expiratory Pressure and Cardiac Index

**DOI:** 10.3389/fped.2015.00005

**Published:** 2015-02-03

**Authors:** Maroun J. Mhanna

**Affiliations:** ^1^Department of Pediatrics, Metro Health Medical Center, Case Western Reserve University, Cleveland, OH, USA

**Keywords:** positive end-expiratory pressure, cardiac index, oxygen transport, critically ill children, infants

Positive end-expiratory pressure (PEEP) is essential in the management of acute respiratory failure (ARF) and acute respiratory distress syndrome (ARDS) in children and adults. Several studies have addressed the pulmonary beneficial effect and cardiovascular impact of an elevated PEEP in adults. However, the impact of an elevated PEEP on the respiratory and cardiovascular systems has not been well elucidated in children. The compliance of the total respiratory system and its components differs between newborn infants, toddlers, children, and adults. For instance, the compliance of the chest wall (as a major component of the total respiratory system) decreases with advancing age (1). Therefore, tolerated elevated levels of PEEP in adults may not be tolerated in children. Similarly, the cardiovascular physiology differs between children and adults. For instance, the systemic vascular resistance (SVR) is higher at baseline in children than adults, and the heart rate (a major component of the cardiac output) is higher in children than adults. Therefore, extrapolating adult physiological studies to children is misleading. Studies addressing the impact of PEEP on the respiratory mechanics and cardiovascular system in children are needed.

Ross and colleagues are to be applauded for studying the impact of high PEEP on the cardiac index (CI) in an animal model with physiologic characteristics that are similar to human infants (2). In their experimental design, they studied nine healthy intubated rhesus monkeys at different levels of PEEP and found a decrease in CI, stroke volume, and oxygen delivery with the increase in PEEP from 5 to 15 cm of H_2_O. The authors attributed the decrease in CI to reduced right ventricular preload as this physiologic mechanism has been well described by previous investigators.

As the authors have mentioned in their discussion, the study has limitations. The small number of animals, and mark variability in response to different levels of PEEP enhance the risk of type I error. However, the authors’ findings in their animal model are consistent with others’ findings in a clinical setting. In a study of 15 critically ill children with ARF, there was a decrease in CI by 15% with the increase in PEEP from 0 to 15 cm of H_2_O (3). However, in that study, the oxygen transport [product of arterial oxygen concentration (CaO_2_) and CI] was not affected by the increasing levels of PEEP. In fact, the PEEP of best oxygen transport was consistent with the PEEP of best CI, and levels of PEEP above that optimal PEEP were associated with a fall in CI and oxygen transport. These clinical findings in pediatric patients with ARF echo the findings of Ross and colleagues. In their animal model, the PEEP of 5 cm of H_2_O was the PEEP of best CI and oxygen delivery, and at higher levels of PEEP the CI and oxygen delivery started to fall. These findings support the validity of their animal model to study the interaction between PEEP and cardiac output. The lack of measurement of respiratory parameters is another limitation of their study. For instance, the authors did not measure tidal volumes, airway pressures, and pleural pressures all of which would have had a significant impact on CI.

In adults with ARDS, open lung ventilation and high PEEP strategies did not decrease mortality in two large multicenter randomized controlled studies (4, 5). However, high PEEP decreased mortality in a subgroup of patients who had an improvement in oxygenation in response to higher PEEP (6). In children, many questions remain answered. Does high PEEP improve the outcome of children with severe ARF and ARDS? And if so, what is considered high PEEP? Especially, since excessive PEEP can be associated with alveolar over distension, ventilation–perfusion mismatch, and impairment of pulmonary vascular resistance.

## Conflict of Interest Statement

The author declares that the research was conducted in the absence of any commercial or financial relationships that could be construed as a potential conflict of interest.
